# Skin Mast Cell Promotion in Random Skin Flaps in Rats using Bone Marrow Mesenchymal Stem Cells and Amniotic Membrane

**DOI:** 10.29252/ibj.22.5.322

**Published:** 2018-09

**Authors:** Farzaneh Chehelcheraghi, Abolfazl Abbaszadeh, Magid Tavafi

**Affiliations:** 1 Department of Anatomical Sciences, School of Medicine, Lorestan University of Medical Sciences, Khorramabad, Iran; 2 Department of Surgery, Lorestan University of Medical Sciences, Khorramabad, Iran

**Keywords:** Angiogenesis, Necrosis, Mast cells, Reconstructive surgical procedures, Surgical flaps

## Abstract

**Background:**

Skin flap procedures are employed in plastic surgery, but failure can lead to necrosis of the flap. Studies have used bone marrow mesenchymal stem cells (BM-MSCs) to improve flap viability. BM-MSCs and acellular amniotic membrane (AAM) have been introduced as alternatives. The objective of this study was to evaluate the effect of BM-MSCs and AAM on mast cells of random skin flaps (RSF) in rats.

**Methods:**

RSFs (80 × 30 mm) were created on 40 rats that were randomly assigned to one of four groups, including (I) AAM, (II) BM-MSCs, (III) BM-MSCs/AAM, and (IV) saline (control). Transplantation was carried out during the procedure (zero day). Flap necrosis was observed on day 7, and skin samples were collected from the transition line of the flap to evaluate the total number and types of mast cells. The development and the total number of mast cells were related to the development of capillaries.

**Results:**

The results of one-way ANOVA indicated that there was no statistically significant difference between the mean numbers of mast cell types for different study groups. However, the difference between the total number of mast cells in the study groups was statistically significant (p = 0.001).

**Conclusion:**

The present study suggests that the use of AAM/BM-MSCs can improve the total number of mast cells and accelerate the growth of capillaries at the transient site in RSFs in rats.

## INTRODUCTION

Surgical skin flaps are frequently used to heal wounds caused by trauma, congenital defects, tumor removal and other issues. Partial skin flap necrosis is a common difficulty in the clinic, particularly on the distal part of the flap. Flap necrosis is caused mainly by inadequate blood perfusion or ischemia-reperfusion. It promotes several damaging alterations in the tissue and vasculature, such as reactive oxygen species and superoxide dismutase activity[1]. Research has been focused on improving blood flow in the flaps, decreasing ischemia, and minimizing necrosis[2,3]. These studies have used bio-scaffolds and stem cell therapy and produced increased blood flow and neoangiogenesis, decreasing the necrotic areas in the skin flaps[4,5].

Stem cells have a high ability for differentiation, and this ability increases during tissue engineering (TE)[6]. Studies have demonstrated that the transplantation of bone marrow mesenchymal stem cells (BM-MSCs) can improve ischemia and flap survival by promoting neovascularization[7]. BM-MSCs promote the secretion of angiogenesis growth factors, such as vascular endothelial growth factor (VEGF) and basic fibroblast growth factor (bFGF), which are critical for neovascularization[7]. However, the squat amount of BM-MSCs in bone marrow has limited its clinical use.

The amniotic membrane (AM) is a form of bio-scaffold derived from the human placenta. AM has the capacity to reduce scarring and inflammation, has anti-microbial properties and improves wound healing. It also assists, as a scaffold, in cell proliferation and differentiation. An AM scaffold is a template of the extracellular matrix (ECM). The goal of AM is the application of biological scaffolds in TE. In addition, AM is a biomaterial that can be easily obtained, produced and transported[8]. AM can be used either with the amniotic epithelium (complete AM) or without it (acellular AM [AMM]). Complete removal of the cellular constituents from AM is essential for the denudation protocol to preserve the mechanical components of the remaining scaffold. A scaffold with the chosen ECM components and low immunogenicity is a preferred matrix for TE[8].

In wounds, mast cells serve as the producer of pro-inflammatory mediators (cytokines). Cytokines can preserve inflammation, vascular modifications and leukocyte penetration[9]. They can generate NGF (nerve growth factor), PDGF (platelet-derived growth factor), VEGF, FGF2, histamine, and tryptase. These growth factors are involved in the proliferation of epithelial cells and fibroblasts. Cytokines can be detected in the early inflammation phase of wound healing[9]. The response of the cytokines to inflammation is stimulation of re-epithelialization, re-angiogenesis and, eventually, collagen and matrix remodeling[9]. After severe inflammation in the first period of wound healing, necrotic tissue is replaced by granulation tissue, which has numerous capillaries[10].

Mast cells are key effector cells in inflammation and wound healing[11]. The aim of the present study was to evaluate morphological changes such as the total number and degranulation of mast cells and the number of type 1, 2, and 3 mast cells in the transitional line using stereological techniques. This experimental study was designed to explore the effects of BM-MSCs and AAM on mast cells in transitional lines in a random rat skin flap model. Wound healing was assessed by evaluating angiogenesis and capillary density.

## MATERIALS AND METHODS

### Cell isolation and labeling

This work was authorized by the Ethics Committee of Lorestan University of Medical Sciences (LUMS.REC.2016.127-5/9/2016). The femur and tibia of Wistar rats were excised and the bone marrow was removed by flushing the hollow bone marrow. This was done using a syringe with a 20-gauge needle filled with DMEM. The collected BM-MSCs were gently pipetted to open cell clusterings and to attain a homogenous cell suspension. The cells were centrifuged at 1200 ×g for 7 min, and the cell pellet was then re-suspended in 3 ml of culture medium. The cell suspension was seeded in 25-cm polypropylene tissue culture flasks with a 5-ml culture medium and preserved in a humid atmosphere with 5% CO_2_ at 37 °C for 15 min. Cultures of the BM-MSCs were examined every three days, and the results were recorded. The cultures were passaged when the BM-MSCs had attained about 80% confluency.

The mesenchymal population was isolated on the basis of its ability to attach to the culture plate[12]. The cell markers on the BM-MSC culture surface to differentiate between media were analyzed using flow cytometry. The cells were marked using field markers for BM-MSC, including CD90, CD105, CD45, and CD34[13]. To place and orient the BM-MSCs at the wound healing flap, the cells were tagged with fluorescent cell tracker 1,1-dioctadyl-3,3,3`,3`-tetramethylindocarbocyanine perchlorate (Dil) dye according to manufacturer instructions. Rat BM-MSCs were fully grown in 75 cm^2^ culture flasks containing fluorescent Dil dye. On the day of labeling, the media was exchanged with 50% fresh BM-MSC media and 50% serum-free media. The cells were collected and re-suspended in serum-free media at a density of 1 × 10^6^/ml. The cells were then labeled with Dil fluorescent cell tracker (cell tracker CM-Dil (C-7000, Molecular Probes Inc., USA) by immersion in 1 ml of a serum-free medium containing 10 µl of Dil at 37 °C for 20 min. The cells were cleaned twice with phosphate buffered saline (PBS) before injection.

### Acellular amniotic membrane preparation

Human AMs were acquired from aseptic sites from 10 women who received elective cesarean sections at Shafa Hospital in Lorestan, Iran. They showed no signs of immunological disorders, hepatitis B, hepatitis C, cytomegalovirus, unsophisticated pregnancies, white-out preterm rupture of membranes, infections, and streptococcus B on vaginal smears or other issues. The AM was eroded three times with antiseptic PBS that included 50 µg/ml of penicillin and 50 µg/ml of streptomycin. The spongy layer was excised and cut into 2.5 × 2.5 cm pieces. The epithelial cells were carefully detached in 0.05% ethylenediaminetetraacetic acid (EDTA; Invitrogen, Germany) at 37 °C for 2 h and were lightly debrided with a cell hand tool under a microscope. The evacuation of epithelial cells was established by exhausting hematoxylin and eosin dye (Sigma-Aldrich, Germany)[14,15].

### Experimental design

**Fig. 1:**
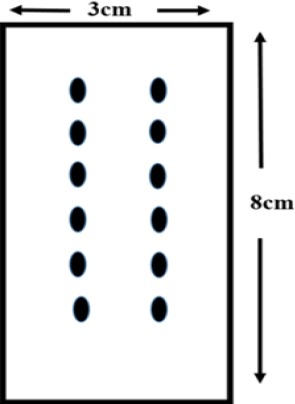
Schematic plot of injection points. Dots indicate the injection points of BM-MSCs.

Forty male Wistar rats, weighing 250 to 350 g, were used in this study. Animal care was provided according to Iranian regulations for research with live animals. All the rats were placed in a prone position and anesthetized with ketamine (50 mg/kg) and xylazine (5 mg/kg). The animals were randomly assigned to one of the four groups as follows: Group 1, dorsal skin flap without MSC injection and AAM transplant receiving 0.5 ml saline injection from the distal to proximal flap in bed; Group 2, dorsal skin flap with an injection of 1 million MSCs in the distal area of the flap; Group 3, dorsal skin flap in which 1 million cells with AAM transplanted flap in bed; Group 4, dorsal skin flap without MSC injection with AAM transplanted flap in bed (Fig. 1).

### Random dorsal skin flap model

After the skin of the rats had been shaved, random skin flaps (RSFs) were prepared, and the full thickness of the skin and skin muscle (panniculus carnosus) were measured. The RSFs were placed at the distal end of the animal on a horizontal line between the iliac crests. The size of the flaps was 30 × 80 mm. After placement, the flaps were immediately renewed using stitched 4.0 nylon thread. The day of surgery was day zero. Directly after surgery, the surface area of the flap was assessed[16].

### Clinical assessments

Assessment was carried out on day 7. Flap viability was determined by clinical (color and capillaries) and histological examinations. The flaps were sliced at the transitional lines and removed and fixed in 10% formalin (Sigma-Aldrich) in PBS at a pH of 7.6[17].

### Histological examinations

**Fig. 2:**
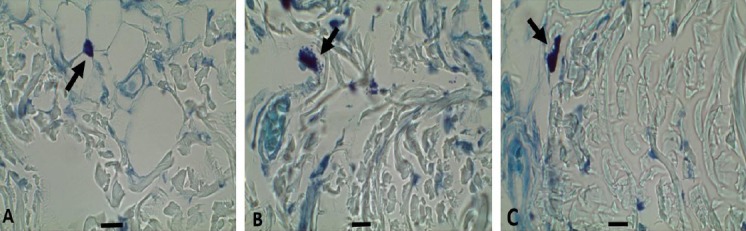
Three steps of mast cell degranulation in the transitional line of the flap seven days after surgery. Tissue sections were stained with toluidine blue. (A) Type 1 mast cell, (B) type 2 mast cell, (C) type 3 mast cell (magnification ×1000; scale bar 10 µm). Arrows show the type of mast cells.

The mast cells in the slices were stained with 10% toluidine blue. There are three types of mast cells[18]. In this work, the formation of the cells (connective tissue) in rats were identified and rested. Considering the grade of formation in each cell, mast cells from the transitional line of the flap were used to epitomize three stages of mast cell suppression. Cells that were entirely stained dark blue were called type 1. Cells in which some granules had been produced (cell outline preserved) were identified as type 2. Cells showing a large and generalized degranulation (total or incomplete impairment of the cell) were identified as type 3 (Fig. 2).

**Fig. 3:**
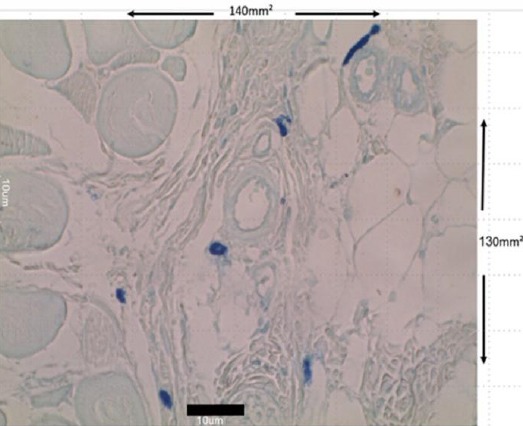
Stereological technique. In this technique, all the elements that are within each frame or adapted to continued lines of the frame would be counted (scale bar 10 µm). In this technique, all the elements that are within each frame or adapted to continued lines of the frame would be counted (scale bar 10 µm).

To accurately assess the number of each type of mast cell in the transitional line of the flap area, a linear zoom (1033.3 mm^2^) was used on the area between necrosis and the healthy zone, with a 100× target. Each group (n = 10) was photographed, and stereological techniques were used to obtain information for 3- dimensional organization based on measures from the two-dimensional sections[19-21]. In this work, 15 mast cell investigation was performed using a specialized cell investigation grid (Fig. 3).

### Immunohistochemistry

Six sample sections of the transitional line area in individual groups were deparaffinized in xylene and rehydrated, then transferred to graded ethanol baths. After coating, the sections were sealed with 3% (v/v) H_2_O_2_ and preserved with 10.2 mM sodium citrate buffer (antigen retrieval) at 95 °C for 20 min. They were then blocked with 5% (w/v) bovine serum albumin and 1% (v/v) Tween-20 in PBS for 10 min. The sections were immersed in VEGF (1:500; PeproTech, Rocky Hill, USA) at 4 °C overnight. Then the sections were immersed in a suitable horseradish peroxidase-conjugated secondary antibody (Santa Cruz Biotechnology, USA) and counterstained with hematoxylin. The number of VEGF-positive blood vessels for each measurement area (2 mm) were assessed. Six random fields of three random sections from each tissue section were used to compute the positive cells[22].

### Statistical analysis

The data was evaluated with SPSS (ver. 16), and all information was analyzed using one-way ANOVA and Tukey’s post hoc comparisons. The results were considered to be significant at *p* < 0.05.

## RESULTS

### Flap survival rate

**Fig. 4:**
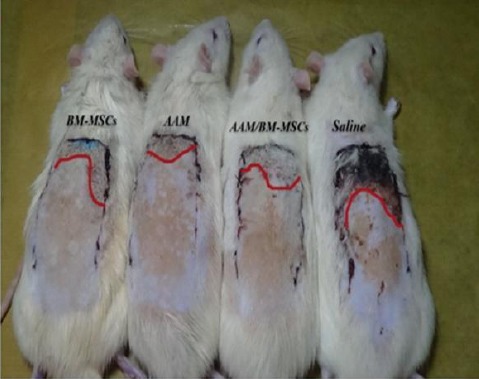
Representive gross view of surviving area.

All animals survived and were accessible for assessment after one week. The flap was unchanged in each animal after microscopic assessment and histologic investigation of viable tissues under a light microscope. Histologically, random tissue samples from the flaps from groups 1, 2, and 3 exhibited flap properties and the inflammatory reactions correlated with wound healing, such as penetration of inflammatory cells (Fig. 4). Occasionally, insignificant necrosis of the border of the flap was observed[4].

### Histology assessments/number of MCs

**Fig. 5:**
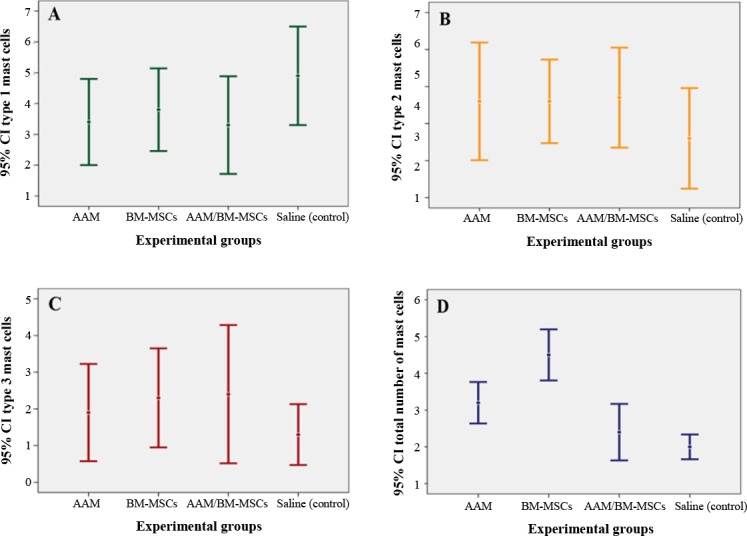
Mean ± SD for the numbers of type 1, 2, and 3 mast cells, and the total number of all types of mast cells in each group in 1033.3-mm^2^ area of full thickness skin of transitional line on day 7, estimated by stereological methods (magnification ×100). Difference between the mean number of mast cells in type 1 (A), type 2 (B) and type 3 (C) in different groups was not statistically significant (p = 0.307, p = 0.536, and p = 0.587, respectively). (D) The mean total number of mast cells in BM-MSCs was higher than AAM group (p = 0.002), AAM/BM-MSCs group (p = 0.001), and the control group (p = 0.001), as well as in AAM, it was higher than AAM/BM-MSCs group (p = 0.044) and the control group (p = 0.003).

The estimated number of all types of mast cells and the total number of mast cells in a 1033.3-mm^2^ area of a transitional line in each group on day 7 after flap surgery are presented in Figure 5. Statistical analysis on day 7, for total number of all types of mast cells, showed significant differences between the study groups (*p* = 0.001). Also, no difference was noted in the numbers of type 1, 2, and 3 mast cells in each group (type 1, *p* = 0.307; type 2, *p* = 0.536; type 3, *p* = 0.587).

### Histology assessments for neovascularization and immunohistochemical analysis

**Fig 6:**
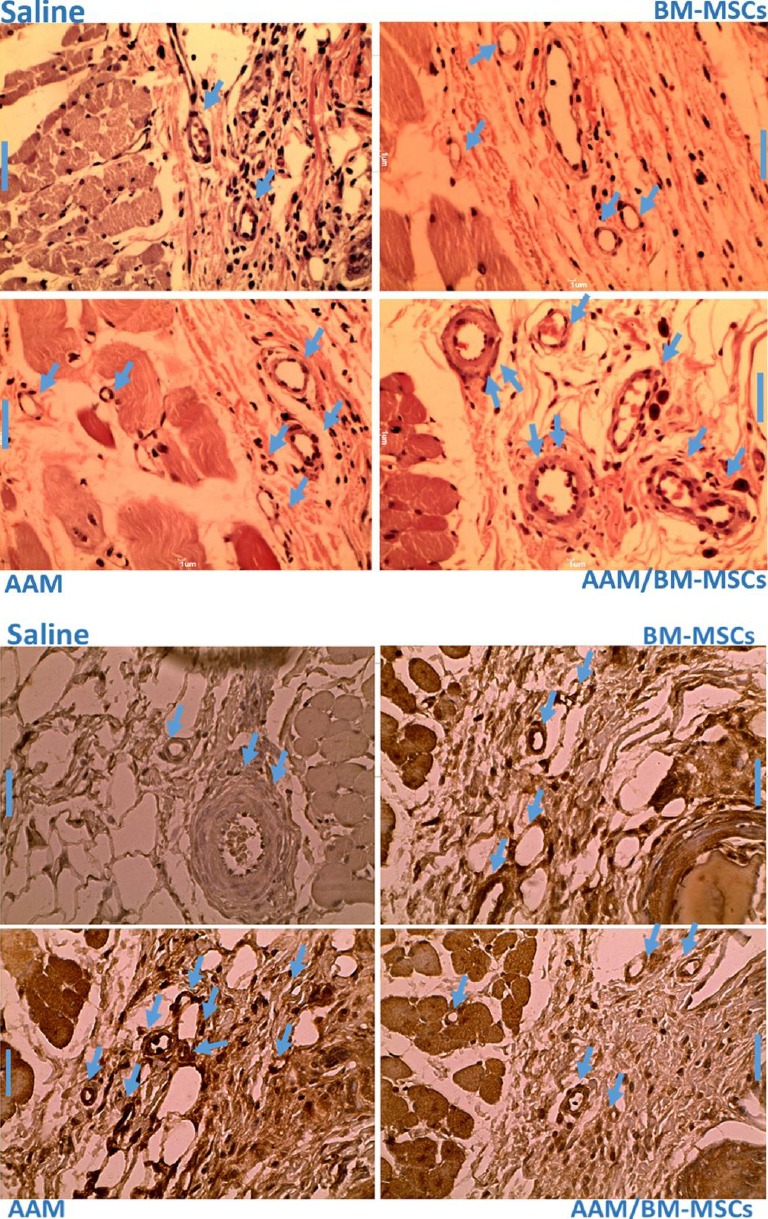
Distribution of blood vessels mean values for the samples. The transitional line of the experimental groups with the original magnification (scale bar 1 µm). Top panel shows H&E staining, and bottom panel shows immunohistochemistry staining. Arrows show vessels.

Neovascularization and immunohistochemical analysis of angiogenesis of the flap were also determined for each group, as shown by overstated amounts of capillary organization in sections along the transitional line (Fig. 6). Qualitative comparisons from immunohistochemical dying revealed that the capillary density was significantly higher in the experimental than contol group (Fig. 6).

### MSC specification by flow cytometry

**Fig 7:**
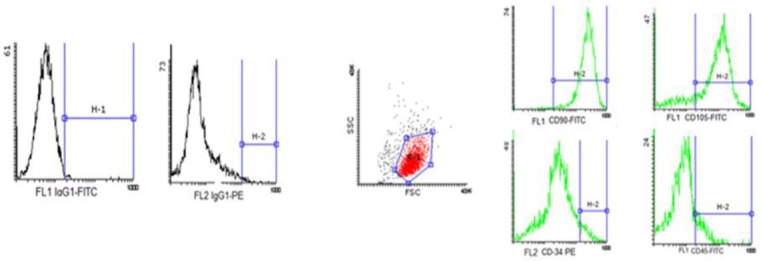
Side scatter channel showing the density plot of BM-MSCs. Characterization of the different surface markers, including CD34, CD45, CD90, and CD105. High expression of CD90 and CD105, low expression of CD34, and no expression of CD45 are shown in the Figure. Also, FL1 and FL2 is control isotope.

Cell surface markers detected by flow cytometry revealed that BM-MSCs strongly expressed CD105 and CD90; however, no expression of CD34 and CD45 was detected (Fig. 7).

### Tracking of transplanted cells

**Fig. 8:**
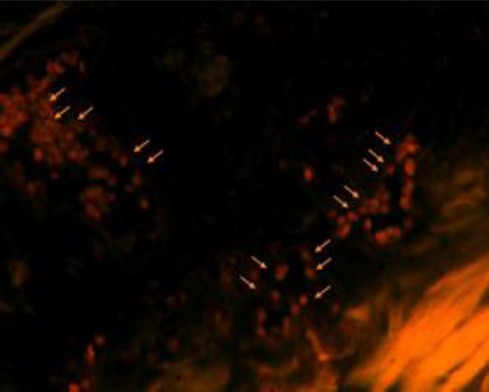
Dil-fluorescence in skin flap. Arrows represent the BMMSCs identified by Dil-fluorescence in skin flap.

On day 7 after surgery, the CM-Dil-labeled BM-MSCs could be still observed in the subcutaneous tissue of the flap. Some of the transplanted BM-MSCs were incorporated into the vascular vessels in the fluorescent slices (Fig. 8).

## DISCUSSION

Studies have revealed that BM-MSCs can affect the speed and the quality of wound healing. A variety of stem cells have been used to treat ischemia, among which BM-MSCs, adipose tissue derived-MSCs and human umbilical cord MSCs are the most studied. The current study seeded BM-MSCs on AAM because of the positive effect of stem cells, especially BM-MSCs, on healing in ischemia and the positive effect of mast cells on angiogenesis in wound healing[13]. The combination of these two features can hypothetically improve the effectiveness of either treatment separately on ischemia of skin flaps and can provide a better prognosis after flap transfer. In the current study, the role of AAM and BM-MSCs on mast cells during ischemia in a dorsal RSF was investigated. Angiogenesis of the skin flap is a crucial and a complex process; it involves the proliferation of endothelial cells and the cooperation of various growth factors[23]. Some studies have found that the expression of CD_31_ and the presence of VEGF leads to angiogenesis and vascular progression. Although pure BM-MSCs and AAM do not have the ability to secret VEGF, the effect of this new management was investigated by measuring the types of mast cells and the vascular density in skin flaps[24]. The gradual changes in appearance of mast cell have been the subject of study aimed at finding the key function of these cells in the dynamic physiology of skin healing. Consideration of the number of mast cells and statistical analysis indicates that the use of BM-MSCs notably expanded the number of mast cells in all experimental categories on day 7. It appears that in the AAM/BM-MSCs group, the number of type 2 mast cells increased compared with control, but the increase was not significant. The number of type 3 mast cells decreased in all categories, but the decrease was not statistically significant. The number of type 3 mast cells decreased on day 7, indicating that AAM/BM-MSCs delayed the conversion of type 2 into type 3 mast cells. In the AAM group, the type 3 mast cells decreased significantly on day 7 compared to the BM-MSCs and AAM/BM-MSCs groups. This event indicates that AAM has a notable effect on decreasing the number of type 3 mast cells, in other words, in converting type 2 mast cells to type 3. It is clear that AAM modulates the production of type 3 mast cells.

Studies have suggested that decreasing the components of inflammation, such as mast cell degranulation, as well as increasing the total number of mast cells can positively affect wound healing[25,26]. This finding indicates that mast cells have similar potential for regenerating flap tissue. Mast cells are sensitive to biochemical signals at sites of injury and inflammation and can be polarized against or in favor of inflammatory phenotypes that can have numerous downstream effects[3,22]. The results of our study showed an increase in type 1 mast cells as well as an increase in the area of necrosis in the control group. The process of converting of type 2 to type 3 mast cells in all treated categories indicates that stages in the control category will change. The process of change was as for the experimental category. It appears that the change type 2 into type 3 mast cells continues in the control and experimental categories.

It was found that although BM-MSCs increased the total number of mast cells in the BM-MSC and AAM/BM-MSC groups compared with the control, this increase is related to type 1 and possibly type 2 mast cells. A previous study has revealed that mast cells contribute to scar tissue during wound repair[17].

When AAM decreases the presence of type 3 mast cells during wound repair, it may prohibit scar formation by its direct and strong influence on fibroblast proliferation[25]. It has been suggested that mast cells contribute to tissue repair through the secretion of soluble factors rather than trans-differentiation[27]. Even though mast cells act as a homeostatic ensemble of skin healing under gradual degranulation, it becomes harmful when it exceeds the threshold of destruction. As a result, the repair stages can be affected by chronic inflammation and changes in the dynamics of proliferation[28].

In the current study, when AAM/BM-MSCs reduced the type 3 mast cells throughout wound healing, it reduced scar formation and influenced fibroblast proliferation. Biological scaffolds such as AM microvascular builder scaffolds are widely used in wound healing[29]. AM acts instead of a scaffold for TE. The ECM elements of the base membrane on the AM contains collagen, fibronectin, laminin, and other proteoglycans, which is remarkable for cell growth. These elements are integrin ligands and are effective in cell adhesion[8].

The study by Younon *et al*.[30] revealed that normal wound healing requires mast cells. The results of another study showed that microdistortion of wounds is a healthy stimulant for the onset of cellular mast-cell activity in a complete wound healing model[9]. Research has demonstrated a connection between the surge in mast cell degranulation and micro-distortion wound therapy and other wound-healing factors[30]. It can be concluded that AAM/BM-MSCs increase the number of type 2 mast cells and cause the development of microvessles. However, further research is needed to determine other mechanisms in which AAM/BM-MSCs suppress the presence of mast cells in inflammation.

## References

[1] Hsueh Y, Wang DH, Huang TC, Chang YJ, Shao WC, Tuan TL, Hughes MW, Wu CC (2016). Novel skin chamber for rat ischemic flap studies in regenerative wound repair. Stem cell research and therapy.

[2] Chehelcheraghi F, Eimani H, Sadraie SH, Torkaman G, Amini A, Shemshadi H, Majd HA (2015). Improved viability of random pattern skin flaps with the use of bone marrow mesenchymal-derived stem cells and chicken embryo extract. Iranian journal of basic medical sciences.

[3] Chehelcheraghi F, Eimani H, Homayoonsadraie S, Torkaman G, Amini A, Alavi Majd H, Shemshadi H (2016). Effects of acellular amniotic membrane matrix and bone marrow-derived mesenchymal stem cells in improving random skin flap survival in rats. Iraiann Red Crescent medical journal.

[4] Rodríguez-Lorenzo A, Arufe MC, de la Fuente A, Fernandez F, Blanco F (2014). Influence of flap prefabrication on seeding of subcutaneously injected mesenchymal stem cells in microvascular beds in rats. Annals of plastic surgery.

[5] Zhang FG, Tang XF (2014). New advances in the mesenchymal stem cells therapy against skin flaps necrosis. World journal of stem cells.

[6] Caplan AI (2007). Adult mesenchymal stem cells for tissue engineering versus regenerative medicine. Journal of cellular physiology.

[7] Uysal CA, Ogawa R, Lu F, Hyakusoku H, Mizuno H (2010). Effect of mesenchymal stem cells on skin graft to flap prefabrication: an experimental study. Annals of plastic surgery.

[8] Niknejad H, Peirovi H, Jorjani M, Ahmadiani A, Ghanavi J, Seifalian AM (2008). Properties of the amniotic membrane for potential use in tissue engineering. European cells and materials.

[9] Babaei S, Bayat M (2012). Effect of pentoxifylline administration on mast cell numbers and degranulation in a diabetic and normoglycemic rat model wound healing. Iranian Red Crescent medical journal.

[10] Fathabadie FF, Bayat M, Amini A, Bayat M, Rezaie F (2013). Effects of pulsed infra-red low level-laser irradiation on mast cells number and degranulation in open skin wound healing of healthy and streptozotocin-induced diabetic rats. Journal of cosmetic and laser therapy.

[11] Nishikori Y, Kakizoe E, Kobayashi Y, Shimoura K, Okunishi H, Dekio S (1998). Skin mast cell promotion of matrix remodeling in burn wound healing in mice: relevance of chymase. Archives of dermatological research.

[12] Galli SJ, Tsai M (2008). Mast cells: versatile regulators of inflammation, tissue remodeling, host defense and homeostasis. Journal of dermatological science.

[13] Khorsandi L, Nejad-Dehbashi F, Ahangarpour A, Hashemitabar M (2015). Three-dimensional differentiation of bone marrow-derived mesenchymal stem cells into insulin-producing cells. Tissue and cell.

[14] Karaoz E, Aksoy A, Ayhan S, Sarıboyacı AE, Kaymaz F, Kasap M (2009). Characterization of mesenchymal stem cells from rat bone marrow: ultrastructural properties, differentiation potential and immunophenotypic markers. Histochemistry and cell biology.

[15] Portmann-Lanz CB, Ochsenbein-Kölble N, Marquardt K, Lüthi U, Zisch A, Zimmermann R (2007). Manufacture of a cell-free amnion matrix scaffold that supports amnion cell outgrowth *in vitro*. Placenta.

[16] Yang L, Shirakata Y, Tokumaru S, Xiuju D, Tohyama M, Hanakawa Y, Hirakawa S, Sayama K, Hashimoto K (2009). Living skin equivalents constructed using human amnions as a matrix. Journal of dermatological science.

[17] Im MJ, Kim YS, Edwards RJ, Hoopes JE, Fenselau A (1992). The effect of bovine basic fibroblast growth factor on skin flap survival in rats. Annals of plastic surgery.

[18] Nishikori Y, Shiota N, Okunishi H (2014). The role of mast cells in cutaneous wound healing in streptozotocin-induced diabetic mice. Archives of dermatological research.

[19] Baddeley AJ, Gundersen HJ, Cruz-Orive LM (1986). Estimation of surface area from vertical sections. Journal of Microscopy.

[20] Gundersen HJ, Bendtsen TF, Korbo L, Marcussen N, Møller A, Nielsen K, Nyengaard JR, Pakkenberg B, Sørensen FB, Vesterby A, West MJ (1988). Some new, simple and efficient stereological methods and their use in pathological research and diagnosis. APMIS.

[21] Amadeu T, Braune A, Mandarim-de-Lacerda C, Porto LC, Desmoulière A, Costa A (2003). Vascularization pattern in hypertrophic scars and keloids: a stereological analysis. Pathology research and practice.

[22] Zhou KJ, Zhang YH, Lin DS, Tao XY, Xu HZ (2016). Effects of calcitriol on random skin flap survival in rats. Scientific reports.

[23] Yang M, Sheng L, Li H, Weng R, Li QF (2010). Improvement of the skin flap survival with the bone marrow-derived mononuclear cells transplantation in a rat model. Microsurgery.

[24] Gruber BL (2003). Mast cells in the pathogenesis of fibrosis. Current rheumatology reports.

[25] Beukelman CJ, Van Den Berg A, Hoekstra M, Uhl R, Reimer K, Mueller S (2008). Anti-inflammatory properties of a liposomal hydrogel with povidone-iodine (Repithel®) for wound healing *in vitro*. Burns.

[26] Craig SS, DeBlois G, Schwartz LB (1986). Mast cells in human keloid, small intestine, and lung by an immuno-peroxidase technique using a murine monoclonal antibody against tryptase. The American journal of pathology.

[27] Noli C, Miolo A (2001). The mast cell in wound healing. Veterinary dermatology.

[28] Zhao M, Zhou J, Li X, Fang T, Dai W, Yin W, Dong J (2011). Repair of bone defect with vascularized tissue engineered bone graft seeded with mesenchymal stem cells in rabbits. Microsurgery.

[29] Weller K, Foitzik K, Paus R, Syska W, Maurer M (2006). Mast cells are required for normal healing of skin wounds in mice. The FASEB journal.

[30] Younan GJ, Heit YI, Dastouri P, Kekhia H, Xing W, Gurish MF (2011). Mast cells are required in the proliferation and remodeling phases of micro deformational wound therapy. Plastic and reconstructive surgery.

